# Carbon-depleted outer core revealed by sound velocity measurements of liquid iron–carbon alloy

**DOI:** 10.1038/ncomms9942

**Published:** 2015-11-24

**Authors:** Yoichi Nakajima, Saori Imada, Kei Hirose, Tetsuya Komabayashi, Haruka Ozawa, Shigehiko Tateno, Satoshi Tsutsui, Yasuhiro Kuwayama, Alfred Q. R. Baron

**Affiliations:** 1 Materials Dynamics Laboratory, RIKEN SPring-8 Center, RIKEN, Hyogo 679-5148, Japan; 2 Department of Earth and Planetary Sciences, Tokyo Institute of Technology, Tokyo 152-8551, Japan; 3 Earth-Life Science Institute, Tokyo Institute of Technology, Tokyo 152-8550, Japan; 4 Laboratory of Ocean-Earth Life Evolution Research, Japan Agency for Marine-Earth Science and Technology, Kanagawa 237-0061, Japan; 5 School of GeoSciences and Centre for Science at Extreme Conditions, University of Edinburgh, Edinburgh EH9 3FE, UK; 6 Institute for Study of the Earth's Interior, Okayama University, Tottori 682-0193, Japan; 7 Research and Utilization Division, SPring-8, Japan Synchrotron Radiation Research Institute, Hyogo 679-5198, Japan; 8 Geodynamics Research Center, Ehime University, Ehime 790-8577, Japan

## Abstract

The relative abundance of light elements in the Earth's core has long been controversial. Recently, the presence of carbon in the core has been emphasized, because the density and sound velocities of the inner core may be consistent with solid Fe_7_C_3_. Here we report the longitudinal wave velocity of liquid Fe_84_C_16_ up to 70 GPa based on inelastic X-ray scattering measurements. We find the velocity to be substantially slower than that of solid iron and Fe_3_C and to be faster than that of liquid iron. The thermodynamic equation of state for liquid Fe_84_C_16_ is also obtained from the velocity data combined with previous density measurements at 1 bar. The longitudinal velocity of the outer core, about 4% faster than that of liquid iron, is consistent with the presence of 4–5 at.% carbon. However, that amount of carbon is too small to account for the outer core density deficit, suggesting that carbon cannot be a predominant light element in the core.

## Abstract (web summary)

The composition of the Earth's core, particularly the light elements present, is not well constrained. Here, the authors report sound velocities of liquid iron-carbon alloy as measured at very high pressures using inelastic X-ray scattering and suggest that carbon cannot be predominant in the outer core.

## Graphical abstract

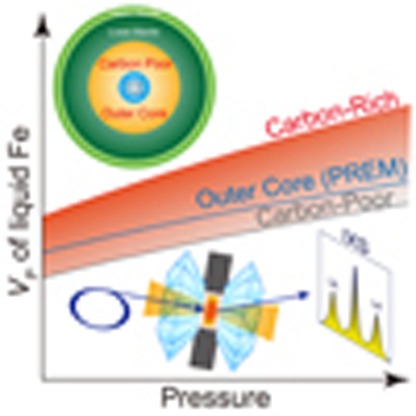

Sound velocity and density are important observational constraints on the chemical composition of the Earth's core. While properties of solid iron alloys have been extensively examined by laboratory studies to core pressures (>136 GPa)^1^,^2^,^3^, little is known for liquid alloys because of experimental difficulties. The core is predominantly molten, and the longitudinal wave (P-wave) velocity of liquid iron alloy is the key to constraining its composition. However, previous static high-pressure and -temperature (*P–T*) measurements of liquid iron alloys were performed only below 10 GPa using large-volume presses^4^,^5^,^6^. Shock wave experiments have been carried out at much higher pressures but only along a specific Hugoniot *P–T* path^7^,^8^.

Carbon is one of the possible light alloying components in the core because of its high cosmic abundance and strong chemical affinity with liquid iron^9^. Its high metal/silicate partition coefficients indicate that thousands of parts per million to several weight percent of carbon could have been incorporated into the core during its formation^9^,^10^,^11^. In addition, recent experimental and theoretical studies^12^,^13^ have suggested that solid Fe_7_C_3_ may explain the properties of the inner core, in particular its high Poisson's ratio^14^,^15^, supporting the presence of carbon in the core.

In this study, we determine the P-wave velocity (*V*_P_) (equivalent to bulk sound velocity, *V*_Φ_, in a liquid) of liquid Fe_84_C_16_ at high *P–T* based on inelastic X-ray scattering (IXS) measurements. Combined with its density data at 1 bar (ref. 16) both velocity and density (*ρ*) profiles of liquid Fe_84_C_16_ along adiabatic compression are obtained. They are compared with seismological observations, indicating that both *V*_P_ and *ρ* in the Earth's outer core are not explained simultaneously by liquid Fe–C.

## Results

### Longitudinal wave velocity measurements

**Figure 1:**
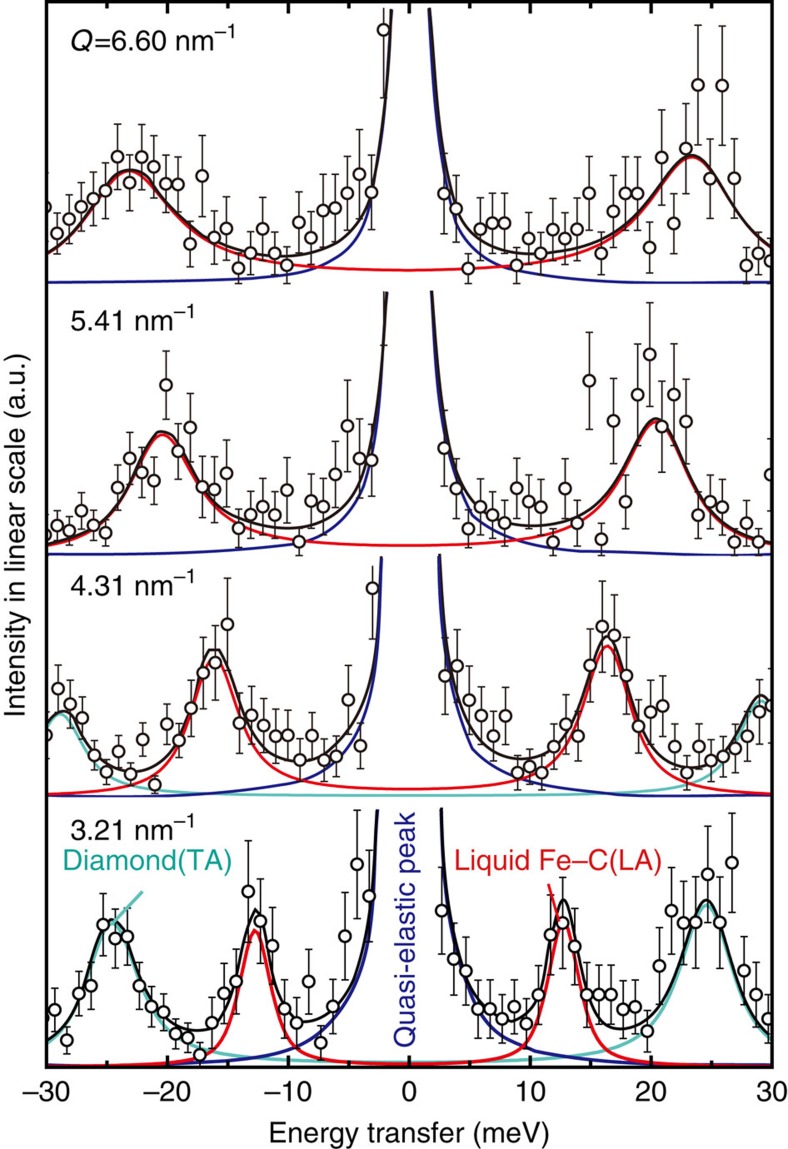
Typical inelastic X-ray scattering spectra. These data were collected at 26 GPa and 2,530 K at momentum transfers *Q*, as indicated. The spectra include three components: a quasi-elastic peak near zero energy transfer (blue), longitudinal acoustic (LA) phonon mode of liquid Fe_84_C_16_ (red), and transverse acoustic (TA) phonon mode of diamond (turquoise).

**Figure 2:**
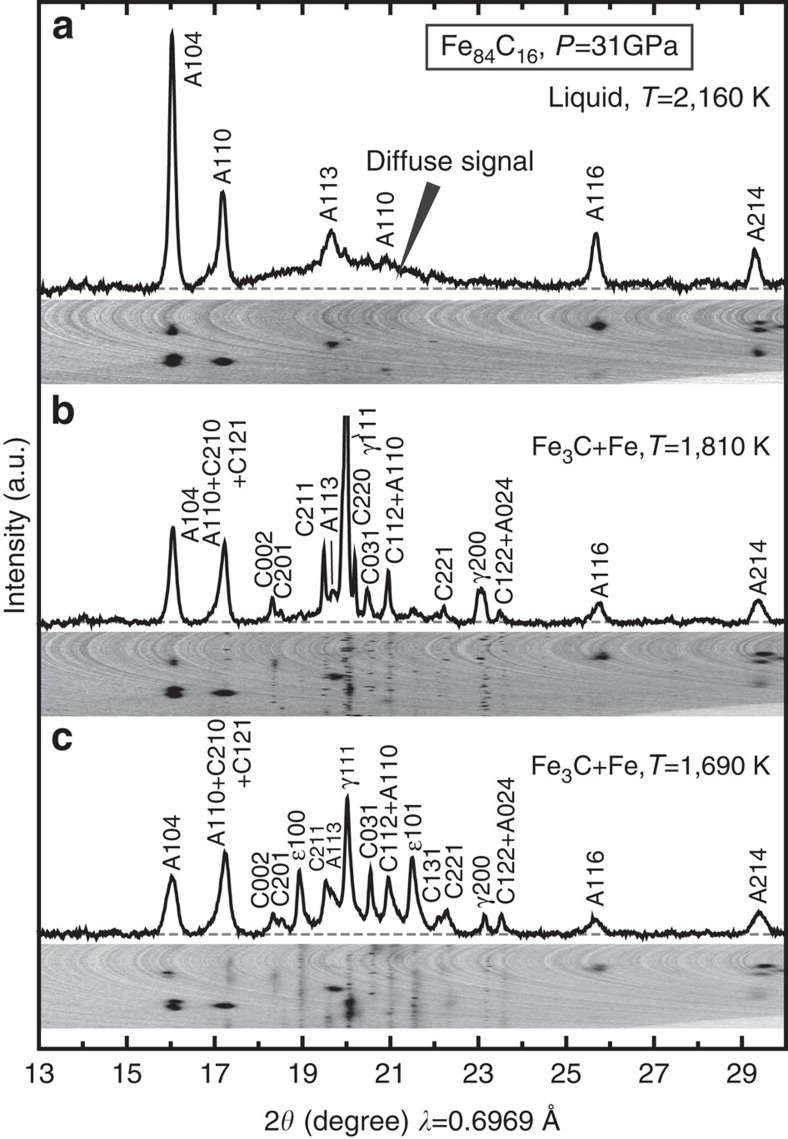
X-ray diffraction spectra before and after melting. They were collected at 2,160 K (**a**), 1,810 K (**b**) and 1,690 K (**c**) during heating at 31 GPa. The starting material was composed of Fe (*ɛ* or *γ*) and Fe_3_C (**c**), and the peaks of Al_2_O_3_ (**a**) were from a thermal insulator. The coexistence of *ɛ*- and *γ*-Fe phases at 1,610 K was due to a sluggish solid–solid phase transition^49^ and the peaks from the *ɛ*-phase were lost at 1,810 K. All sample peaks disappeared between 1,810 and 2,160 K. In addition, the background was enhanced slightly, indicating a diffuse scattering signal from a liquid sample.

We collected the high-resolution IXS spectra from liquid Fe_84_C_16_ (4.0±0.3 wt.% carbon) at static high *P–T* using both resistance- and laser-heated diamond-anvil cells (Methods; Fig. 1). The starting material was synthesized beforehand as a mixture of fine-grained Fe and Fe_3_C at 5 GPa and 1,623 K in a multi-anvil apparatus. Experimental *P–T* conditions were well above the eutectic temperature in the Fe–Fe_3_C binary system (Supplementary Fig. 1). The carbon concentration in the eutectic liquid is known to be 3.8–4.3 wt.% at 1 bar to 20 GPa (ref. 17), almost identical to the composition of our sample. Above 20 GPa, we heated the sample to temperatures comparable or higher than the melting temperature of Fe_3_C, a liquidus phase in the pressure range explored, assuring a fully molten sample. The molten state of the specimen was carefully confirmed, before and after the IXS measurements, by the absence of diffraction peaks from the sample (Fig. 2). We sometimes, depending on a sample volume, were also able to observe the diffuse diffraction signal typical of a liquid.

**Figure 3:**
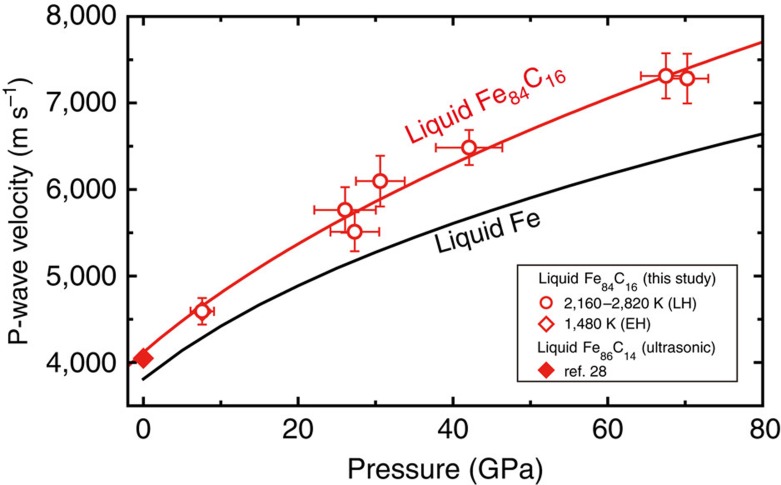
Compressional wave velocity of liquid Fe_84_C_16_. Open circles, obtained by laser-heated DAC; open diamond, by external-resistance-heated DAC. The data at 1 bar is from ultrasonic measurements^28^ (closed diamond). The red curve represents a thermodynamical fitting result for liquid Fe_84_C_16_, compared with the velocity of liquid Fe (black curve)^8^.

**Figure 4:**
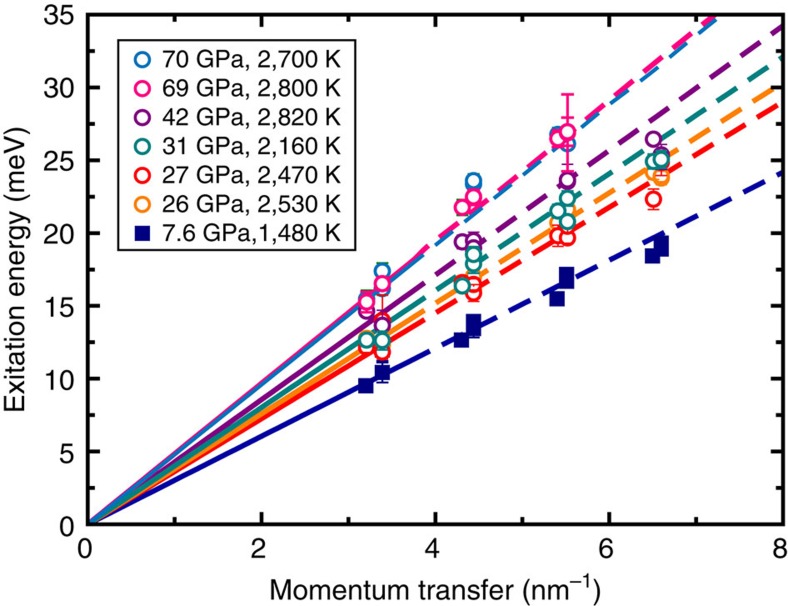
Longitudinal acoustic phonon dispersion of liquid Fe_84_C_16_. The dispersion data were obtained at pressures from 7.6 to 70 GPa. Only data collected with the low momentum transfer (<3.5 nm^−1^) were used to determine the velocity to avoid possible anomalous dispersion for liquid (see Methods).

**Figure 5:**
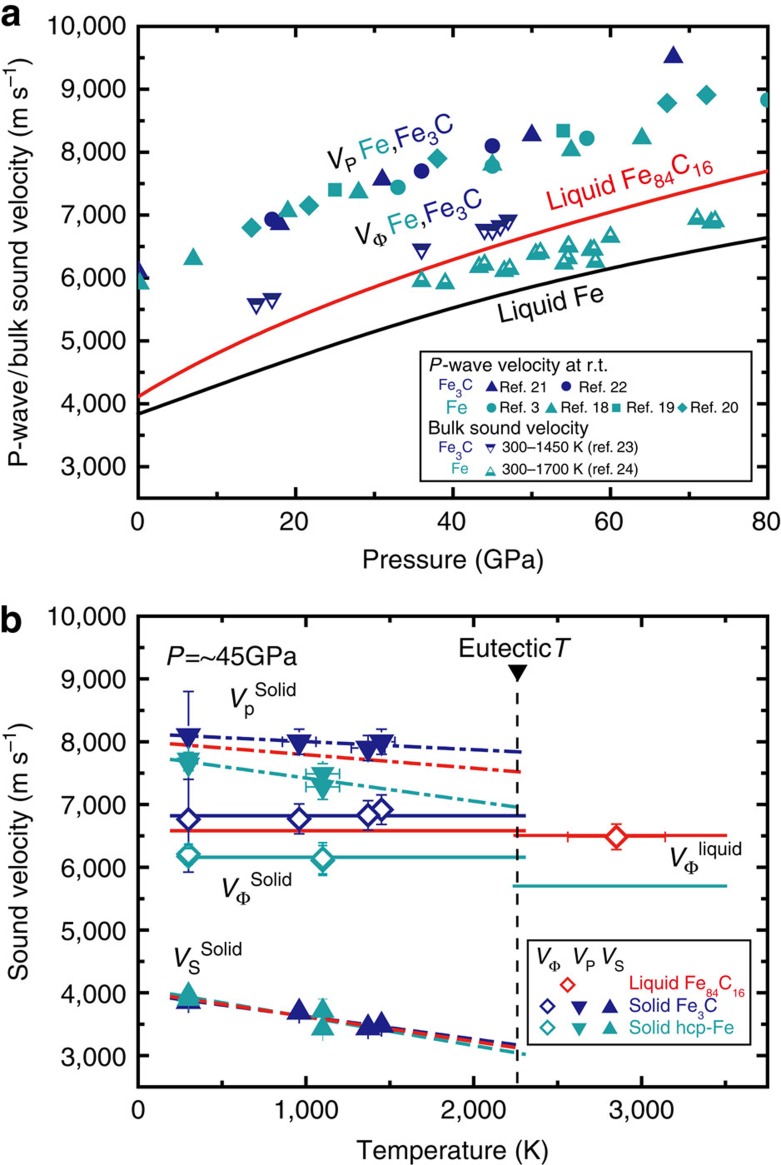
Comparison of velocities between liquid and solid Fe–C alloys. (**a**), P-wave velocities (*V*_P_) and bulk sound velocities (*V*_Φ_) of solid Fe (turquoise)^3^,^18^,^19^,^20^,^24^ and Fe_3_C (blue)^21^,^22^,^23^ were determined by previous IXS and nuclear inelastic scattering (NIS) measurements. The *V*_P_ for liquid Fe is from shock-wave study^8^. (**b**), Temperature effects on the sound velocities of Fe–C alloys at ∼45 GPa. The *V*_P_ (= *V*_Φ_) of liquid Fe_84_C_16_ and liquid Fe is from the present work at 42 GPa and shock-wave data^8^, respectively. The *V*_P_, shear velocity (*V*_S_), and *V*_Φ_ for solid Fe and Fe_3_C were reported by NIS measurements^23^,^24^. Red lines for fictional solid Fe_84_C_16_ are estimated from a linear relationship between Fe and Fe_3_C.

The *V*_P_ of liquid Fe_84_C_16_ was determined between 7.6 and 70 GPa (Fig. 3 and Supplementary Table 1) from dispersion curves for a range of momentum transfer (Fig. 4). It was found to be 15–30% smaller than that of solid Fe (refs 3, 18, 19, 20) and Fe_3_C (refs 21, 22, 23; note that a starting material in the present experiments was a mixture of these solid phases) (Fig. 5), confirming that we measured a liquid sample. The velocities of a fictive solid Fe_84_C_16_ alloy are also estimated assuming a linear velocity change between Fe (ref. 24) and Fe_3_C (ref. 23) indicating that *V*_P_ drops by 13% upon melting at 2,300 K, a eutectic temperature at 45 GPa (ref. 17). Such a velocity change is comparable to that expected for pure Fe. The difference in *V*_Φ_ between solid and liquid Fe_84_C_16_ is very small (1.8%). On the other hand, the *V*_P_ of our liquid Fe_84_C_16_ sample is 3–14% faster at 8–70 GPa than that of liquid Fe determined by shock-wave study^8^ (Fig. 3).

Earlier ultrasonic measurements performed below 10 GPa reported a change in *V*_P_ by <2–3% per 1,000 K for liquid Fe–S alloys^4^,^5^. Theoretical calculations^25^,^26^,^27^ and shock compression data^8^ on liquid Fe and Fe–S alloy demonstrated even smaller effects above 100 GPa (<0.5% by 1,000 K). It is therefore very likely that the *V*_P_ of liquid Fe_84_C_16_ is also not sensitive to temperature with the temperature effect much smaller than the uncertainty in the present velocity determinations (±3%).

### Thermodynamical equation of state

*V*_P_ of a liquid can be described using the Murnaghan equation of state^4^ (Methods) as;


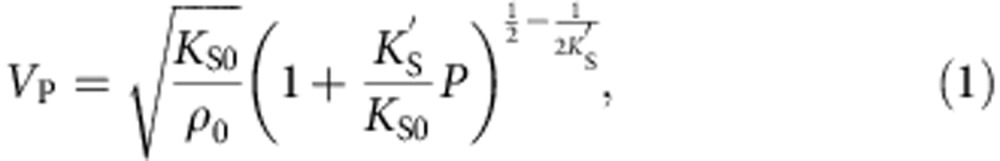


where *K*_S_ and *K′*_S_ are adiabatic bulk modulus and its pressure derivative, respectively (zero subscripts denote values at 1 bar and *T*=*T*_0_). Here, consistent with the discussion above, we neglect the temperature dependence of our *V*_P_ data, while *ρ*_0_ is taken to be temperature dependent^16^ (Methods). We fit equation (1) to our *P*–*V*_P_ data for liquid Fe_84_C_16_ and find *K*_S0_=110±9 GPa and *K*′_S_=5.14±0.30 when *T*_0_=2,500 K (Supplementary Table 2 and Supplementary Fig. 2). The choice of *T*_0_ and, accordingly, the variation in *ρ*_0_ practically changed *K*_S0_ and *K*′_S0_ as (∂*K*_S0_/∂*T*)=−9.4 × 10^−3^ GPa K^−1^ and (∂*K*′_S0_/∂*T*) =−2.7 × 10^−4^ K^−1^. Our value for *K*_S0_ is similar to that for liquid iron^8^ but for *K*′_S_ is higher than that for pure iron, *K*′_S_=4.7. This suggests that liquid Fe_84_C_16_ becomes progressively stiffer than liquid Fe with increasing pressure. We also found *V*_P0_=4,121±177 m s^−1^ for liquid Fe_84_C_16_ from *K*_S0_ and *ρ*_0_, in good agreement with a previous study^28^ of liquid Fe_86_C_14_ at 1 bar (4,050 m s^−1^) and faster than *V*_P0_=3,860 m s^−1^ for liquid Fe (ref. 8).

**Figure 6:**
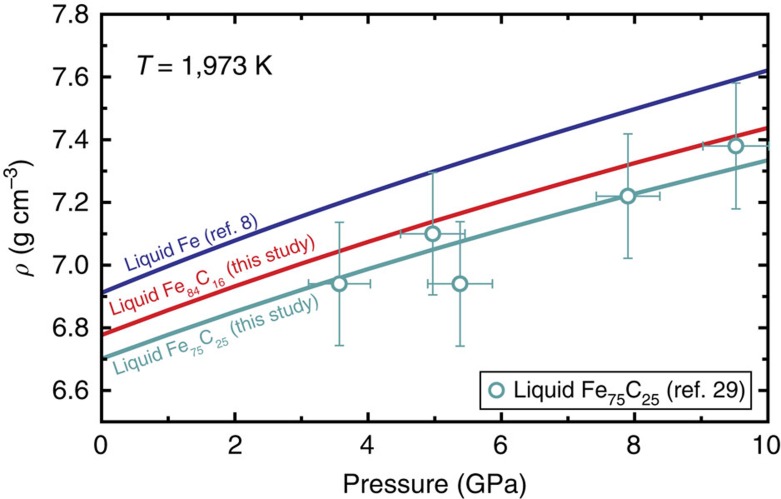
Comparison with previous density measurements. Blue and red curves demonstrate calculated densities at 1,973 K for liquid Fe (ref. 8) and Fe_84_C_16_ (present study). The density of liquid Fe_75_C_25_ (turquoise curve) is estimated assuming linear compositional dependence between pure Fe and Fe_75_C_25_, which shows good agreement with the previous measurements at 1,973 K (ref. 29).

To compare the present results with earlier density measurements of liquid Fe–C alloys at high pressure^29^,^30^ the isothermal bulk modulus for liquid Fe_84_C_16_ is estimated to be *K*_T0_=100 (82) GPa at 1,500 K (2,500 K) from our determination of *K*_S_ combined with Grüneisen parameter *γ*_0_=1.74 (ref. 8) and thermal expansion coefficient^16^ (Methods). When applying *C*_P_/*C*_V_=1.125 at 1,820 K for liquid Fe_86_C_14_ derived from theoretical calculations^31^, *K*_T0_=106–98 GPa is obtained at the same temperature range. These *K*_T0_ values for liquid Fe_84_C_16_ are similar to *K*_T0_=95–63 GPa for liquid Fe at 1,500–2,500 K (ref. 8) On the other hand, they are significantly larger than *K*_T0_=55.4 GPa for liquid Fe_86_C_14_ at 1,500 K and *K*_T0_=50 GPa for liquid Fe_75_C_25_ at 1,973 K from previous density measurements^29^,^30^. However, the calculated density for Fe_84_C_16_ using the present EoS are in reasonable agreement with the previous density measurements of Fe_75_C_25_ (ref. 29) (Fig. 6). The disagreement of elastic parameters with such earlier experiments may be attributed either to the limited pressure range of the previous density determinations, or to a different structure or magnetic (or electronic) change in the state of the liquid Fe–C at low pressure, as has been suggested from the change in compressional behaviour of liquid Fe_78_C_22_ around 5 GPa (ref. 6). Our data were collected above 7.6 GPa, so that the physical properties of liquid Fe–C obtained here should be more applicable to the Earth's core.

*ρ* of liquid Fe_84_C_16_ is then given, using the elastic parameters determined above, by;


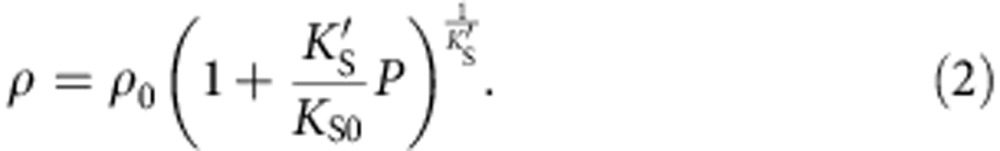


**Figure 7:**
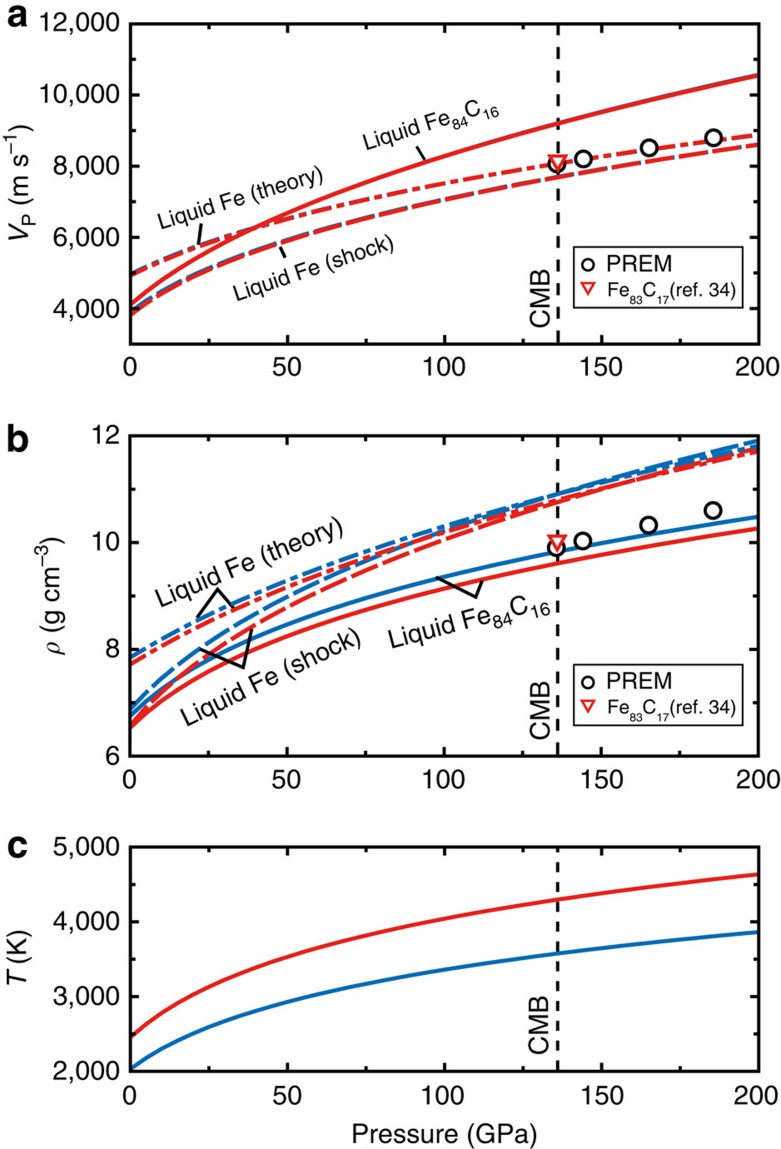
Velocity and density of liquid Fe_84_C_16_ extrapolated to core pressures. The P-wave velocity (**a**) and density (**b**) profiles of liquid Fe_84_C_16_ are calculated along two adiabatic temperature curves (**c**) of 3,600 K (blue) and 4,300 K (red) at the CMB. Those for liquid Fe are from shock-compression experiments^8^ and theoretical calculations^26^. The velocity and density for liquid Fe_83_C_17_ at the CMB (4,300 K) are by theory^34^. PREM denotes seismologically deduced Preliminary Reference Earth model^35^.

Equations (1) and (2) give the *V*_P_ and *ρ* profiles for adiabatic compression (Methods), assuming *γ*_0_=1.74, the same as that of liquid Fe (ref. 8) (Fig. 7). We find *V*_P_=9,200 m s^−1^ and *ρ*=9.82–9.61 g cm^−3^ at the core-mantle boundary (CMB) for *T*_CMB_=3,600–4,300 K (refs 32, 33) This indicates that *V*_P_ of liquid Fe_84_C_16_ is 19.6% faster than that of liquid Fe at the CMB^8^, implying that the addition of 1 at.% carbon increases the *V*_P_ of liquid Fe by 1.2%. The extrapolation of the present experimental data using the Murnaghan equation of state may overestimate the *V*_P_ by 2−4% at the CMB (Supplementary Note 1 and Supplementary Fig. 3), but, even if this is the case, 1 at.% carbon enhances the *V*_P_ of liquid Fe by as large as 0.8%. Indeed, the effect of carbon is much larger than a recent theoretical prediction of only 0.2% increase in velocity per 1 at.% carbon at 136 GPa (ref. 34). On the other hand, our data show that the incorporation of 1 at.% carbon reduces the density of liquid Fe by 0.6–0.7%, while theory suggested only 0.3% density reduction by 1 at.% carbon^34^.

## Discussion

**Figure 8:**
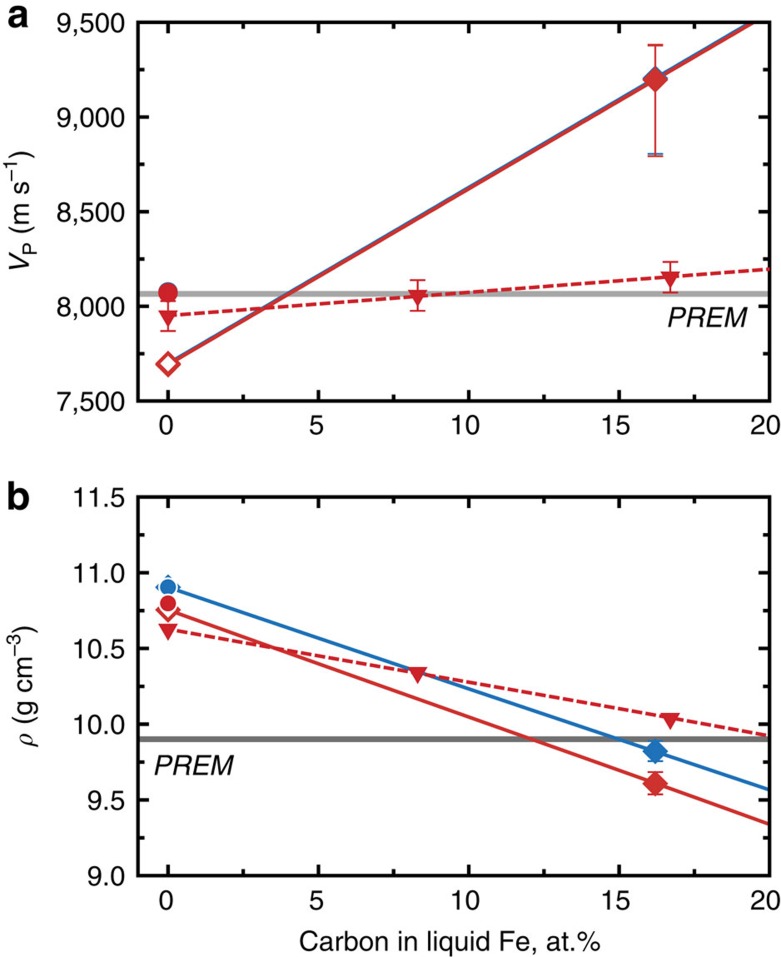
Effect of carbon on the velocity and density of liquid Fe at 136 GPa. (**a**) Velocity and (**b**) density for liquid Fe_84_C_16_ at *T*_CMB_=4,300 K (red) and 3,600 K (blue). Present results (closed diamonds, solid curves) are compared with theoretical calculations^34^ (triangles, broken curve). The data for pure Fe are from shock compression study^8^ (open diamonds) and theoretical calculations^26^ (closed circles). PREM denotes seismological observations^35^ at the CMB.

We now compare the sound velocity and density of liquid Fe_84_C_16_ and liquid Fe with the seismologically based PREM model^35^ for the outer core (Fig. 8). The *V*_P_ and *ρ* of liquid Fe are 4.6% slower and 10.1–8.6% denser, respectively, than the PREM at the CMB (3,600–4,300 K). To match the PREM values, considering the uncertainty of data extrapolation to higher pressures (Supplementary Note 1), only 5.2–4.0 at.% (1.2–0.9 wt.%) carbon is required to match the velocity, whereas 15.4–12.0 at.% (3.8–2.9 wt.%) carbon is necessary to account for the density. Therefore, carbon cannot be a predominant light element in the outer core.

These results suggest there is <5.2 at.% (1.2 wt.%) carbon in the outer core, consistent with the previous cosmochemical and geochemical arguments. In particular, the silicate portion of the Earth exhibits much higher ^13^C/^12^C isotopic ratio than that of Mars, Vesta and chondrite meteorites, as may be attributed to a strong enrichment of ^12^C in core-forming metals^9^. The carbon isotopic fractionation that occurred during continuous core-formation process proposed previously^36^,^37^ will give a reasonable ^13^C/^12^C ratio in the silicate Earth, and yields 1 wt.% carbon in the core^9^. In addition, Wood *et al.*^9^ demonstrated that carbon strongly affects the chemical activity of Mo and W in liquid metal, so that their abundance in the mantle can be explained by partitioning between silicate melt and core-forming metal with ∼0.6 wt.% carbon. It has been repeatedly suggested that the inner core may be composed of Fe_7_C_3_, which accounts for high Poisson's ratio observed^14^,^15^. The crystallization of solid Fe_7_C_3_ from a liquid outer core with <1.2 wt.% carbon may still be possible if sulfur is also included in the core^38^.

## Methods

### High *P–T* generation

Molten Fe–C alloy was obtained at high *P–T* in an external-resistance-heated (EH) or laser-heated (LH) diamond-anvil cell (DAC; Supplementary Table 1) using facilities installed at SPring-8. A disc of pre-synthesized Fe_84_C_16_ sample, 20–25 μm thick and 100–120 μm in diameter, was loaded into a hole of a rhenium gasket, together with two 12–17 μm thick single-crystal Al_2_O_3_ sapphire discs that served as both thermal and chemical insulators. The sample was compressed with 300 μm culet diamond anvils to a pressure of interest before heating.

In LH-DAC experiments, the sample was heated at high pressure from both sides by using two 100 W single-mode Yb fibre lasers (YLR-100-AC, IPG Photonics Corp.). The Gaussian-type energy distribution of the laser beam was converted into flat-top one with a refractive beam shaper (GBS-NIR-H3, Newport Corp.). A typical laser spot was 50–70 μm in diameter on the sample, much larger than X-ray beam size (∼17 μm). We determined temperature by a spetroradiometric method, and its variations within the area irradiated by X-rays and fluctuations during IXS measurements were <±10%. The pressure was obtained from the equation of state for Fe_3_C (ref. 39) from the lattice constant observed before melting at 1,800–2,500 K. Its error was derived from uncertainties in both temperature and the volume of Fe_3_C. A typical image of a sample recovered after the laser heating experiment at 70 GPa and 2,700 K is given in Supplementary Fig. 4.

Only run #FeC08 was conducted in an EH-DAC. The whole sample was homogeneously heated by a platinum-resistance heater placed around the diamonds. The temperature was obtained with a Pt-Rh (type-R) thermocouple whose junction was in contact with the diamond near a sample chamber. The temperature uncertainty was <20 K. We determined the pressure based on the Raman shift of a diamond anvil^40^ before heating at 300 K, whose uncertainty may be as much as ±20%.

### IXS measurements

The sound velocity of liquid Fe–C alloy was determined in the DAC by high-resolution IXS spectroscopy at the beamline BL35XU, SPring-8 (ref. 41). Both LH- and EH-DACs were placed into vacuum chambers to minimize background scattering by air. The measurements were carried out with ∼2.8 meV energy resolution using Si (999) backscattering geometry at 17.79 keV. The experimental energy resolutions were determined using scattering from Polymethyl–methacrylate. The incident X-ray beam was focused to about 17 μm size (full width at half maximum) in both horizontal and vertical directions by using Kirkpatrick–Baez mirrors^42^. The X-ray beam size was much smaller than heated area (50–70 μm for LH-DAC). Scattered photons were collected by an array of 12 spherical Si analyzers leading to 12 independent spectra at momentum transfers (*Q*) between 3.2 and 6.6 nm^−1^ with a resolution Δ*Q* ∼0.45 nm^−1^ (full width) that was set by slits in front of the analyzer array. The energy transfer range of ±30 (or −10 to ±30) meV was scanned for 1–3 h. Before and after IXS data collections, sample melting was confirmed by X-ray diffraction data (Fig. 2) that was collected, *in situ*, by switching a detector to a flat panel area detector (C9732DK, Hamamatsu Photonics K.K.)^43^.

The IXS spectra included three (sometimes five) peaks (Fig. 1) of Stokes and anti-Stokes components of the longitudinal acoustic (LA) phonon mode from the sample (sometimes also from a diamond), and a quasi-elastic contribution near zero energy transfer. These spectra were fitted with the damped harmonic oscillator (DHO) mode^44^ for acoustic phonon modes and with Lorenzian function for quasi-elastic peaks convolved by experimental resolution function. The DHO model function can be described as;


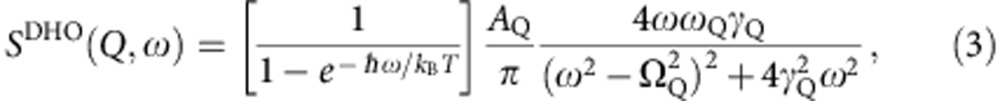


where *A*_Q_, *Γ*_Q_, Ω_Q_, *k*_B_ and *ħ* are the amplitude, width, and energy of inelastic modes, Boltzmann constant and Planck constant, respectively. In the fitting, temperature *T* was fixed at a sample temperature obtained by a spetroradiometric method or a thermocouple. The excitation energy modes appearing at both Stokes and anti-Stokes sides correspond to the phonon creation and annihilation, respectively. With increasing temperature, as given by the Bose function in equation (3), the intensities of such Stokes and anti-Stokes peaks become similar to each other. A symmetric shape of the present IXS spectra therefore assures that the IXS signals originated from a high-temperature area.

The peak at a finite energy transfer gives the frequency of each mode (Fig. 1). The excitation energies for the LA phonon mode of liquid Fe_84_C_16_ obtained in a pressure range of 7.6–70 GPa are plotted as a function of momentum transfer (*Q*) in Fig. 4. The compressional sound wave or P-wave velocity (*V*_P_) corresponds to the long-wavelength LA velocity at *Q*→0 limits;


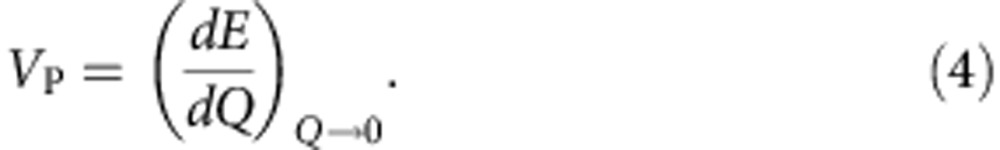


We made a linear fit to the data obtained at low *Q* below 3.5 nm^−1^ to determine the P-wave velocity (Supplementary Table 1), because positive dispersion can appear at higher *Q*>>3 nm^−1^ (ref. 45). For comparison, the results based on a sine-curve fit to all *Q*-range data, as is usually applied for polycrystalline samples in similar high-pressure IXS measurements^46^, are also given in Supplementary Table 1. In general, the error bars of the two determinations of *V*_P_ overlap, though the sine fit to large *Q* does give slightly larger *V*_P_, as would qualitatively be expected from previous measurements on liquid iron^47^.

### Equation of state for liquid Fe_84_C_16_

We constructed an equation of state (EoS) for liquid Fe_84_C_16_ to extrapolate the present *V*_P_ data and to estimate its density at the core pressure range. *V*_P_ of liquid can be written as;


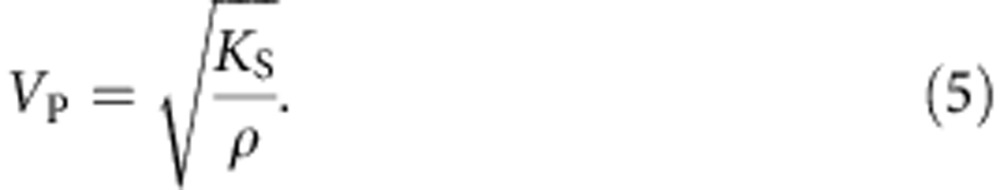


The pressure dependence of *K*_S_ is assumed to be


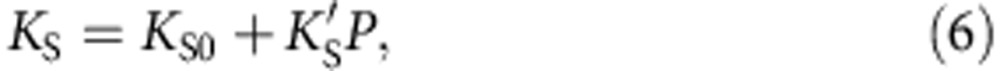


where *K′*_S_ is the pressure derivative of *K*_S_ and pressure and subscript zero indicates a value at 1 bar. The adiabatic Murnaghan EoS can be described as (for example, ref. 4);


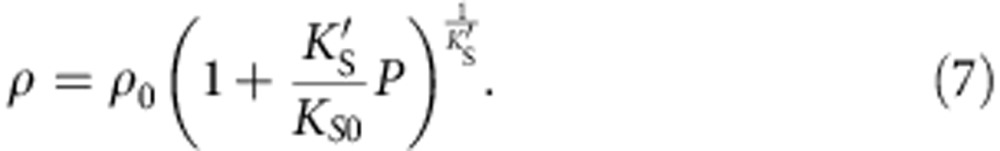


Equation (5) is thus rewritten as;


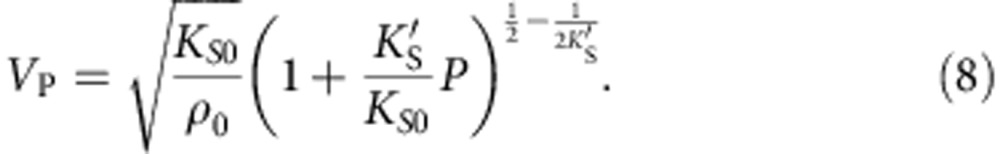


The temperature effect on *ρ*_0_ can be expressed by;


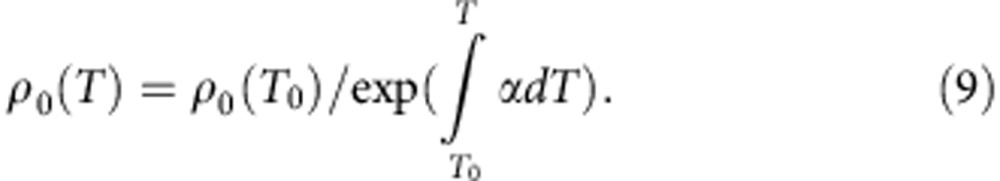


The thermal expansion coefficient *α* is also dependent on temperature as;


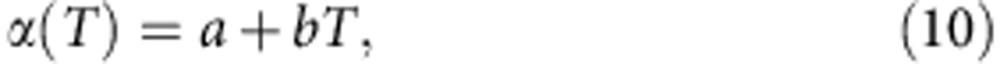


where *a* and *b* are constants. Previous density measurements^16^ of liquid Fe–C alloys at 1 bar give *a*=6.424 × 10^−5^ K^−1^ and *b*=0.606 × 10^−8^ K^−2^ for liquid Fe_84_C_16_ using *ρ*_0_=6.505 g cm^−3^ at *T*_0_=2,500 K as a reference. The result of fitting equation (8) to the present *P*−*V*_P_ data is given in Fig. 3.

### Isothermal bulk modulus

We estimate isothermal bulk modulus *K*_T_ from isentropic bulk modulus *K*_S_ in two ways. The relationship between these two is described as follows;


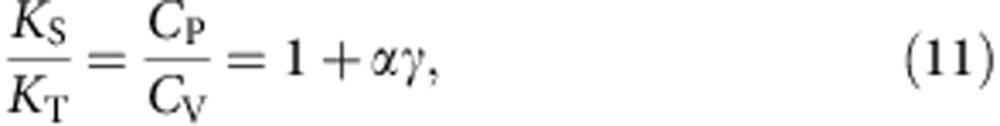


where *C*_P_ and *C*_V_ are heat capacities at constant pressure and volume, respectively. Although *γ* for liquid Fe–C alloys is not known, *γ*_0_=1.74 has been reported for liquid Fe at 1 bar and 1,811 K (ref. 8) It is close to 1.58 for liquid Fe_90_O_8_S_2_ estimated from the shock compression data set^48^.

### Extrapolation of present data to core pressures

With the EoSs determined above (equations (7) and (8)), we extrapolate the P-wave velocity and density of liquid Fe_84_C_16_ to the core pressure range along adiabatic compression, in which temperature is given by;


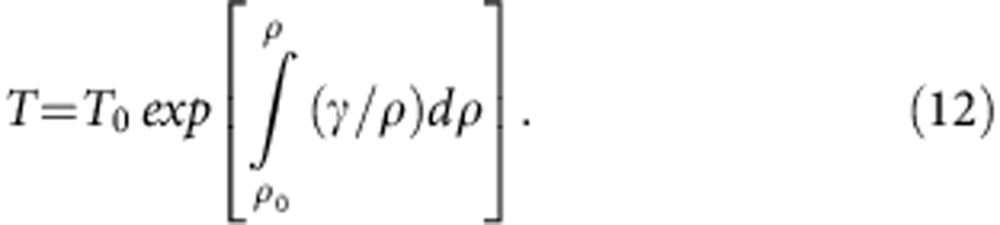


Assuming *γ*=*γ*_0_ × (*ρ*_0_/*ρ*), temperature is simply represented as;


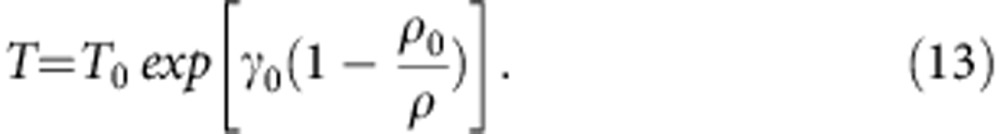


*γ*_0_ is fixed at 1.74 previously obtained for liquid Fe (ref. 8). Using the temperature dependence of *K*_S0_ and *ρ*_0_ shown above, we calculate density, velocity and temperature profiles along adiabatic compression with various reference temperatures at the CMB. The adiabatic compression profiles of liquid Fe_84_C_16_ for the low (*T*_0_=2,045 K and *T*_CMB_=3,600 K)^32^ and high (*T*_0_=2,457 K and *T*_CMB_=4,300 K)^33^ temperature cases are calculated in Fig. 7.

## Additional information

**How to cite this article:** Nakajima, Y. *et al.* Carbon-depleted outer core revealed by sound velocity measurements of liquid iron–carbon alloy. *Nat. Commun.* 6:8942 doi: 10.1038/ncomms9942 (2015).

## Supplementary Material

Supplementary Information Supplementary Figures 1-4, Supplementary Tables 1-2, Supplementary Note 1 and Supplementary References
