# Supplementary material for: Carbon-depleted outer core revealed by sound velocity measurements of liquid iron–carbon alloy
Source: Nat Commun. 2015 Nov 24;6:8942. doi: 10.1038/ncomms9942 (PMC4673837; doi:10.1038/ncomms9942)
Supplement: Supplementary Information — Supplementary Figures 1-4, Supplementary Tables 1-2, Supplementary Note 1 and Supplementary References [file ncomms9942-s1.pdf]

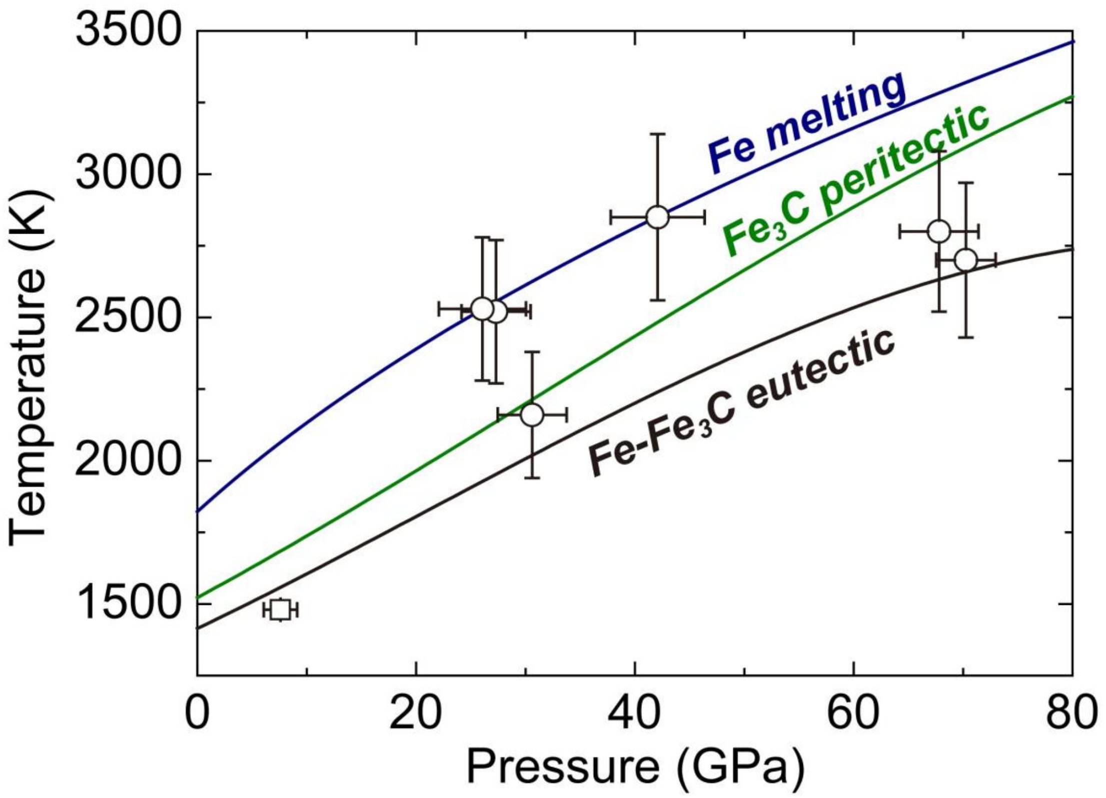

Supplementary Figure 1 | Pressure and temperature conditions of the present experiments. Circle (LH-DAC) and square (EH-DAC) symbols denote the present experimental conditions. Curves show the melting temperatures of pure Fe (blue)[1] and Fe_3C (green)[2] and the eutectic temperature in the Fe-Fe_3C binary system (black)[2].

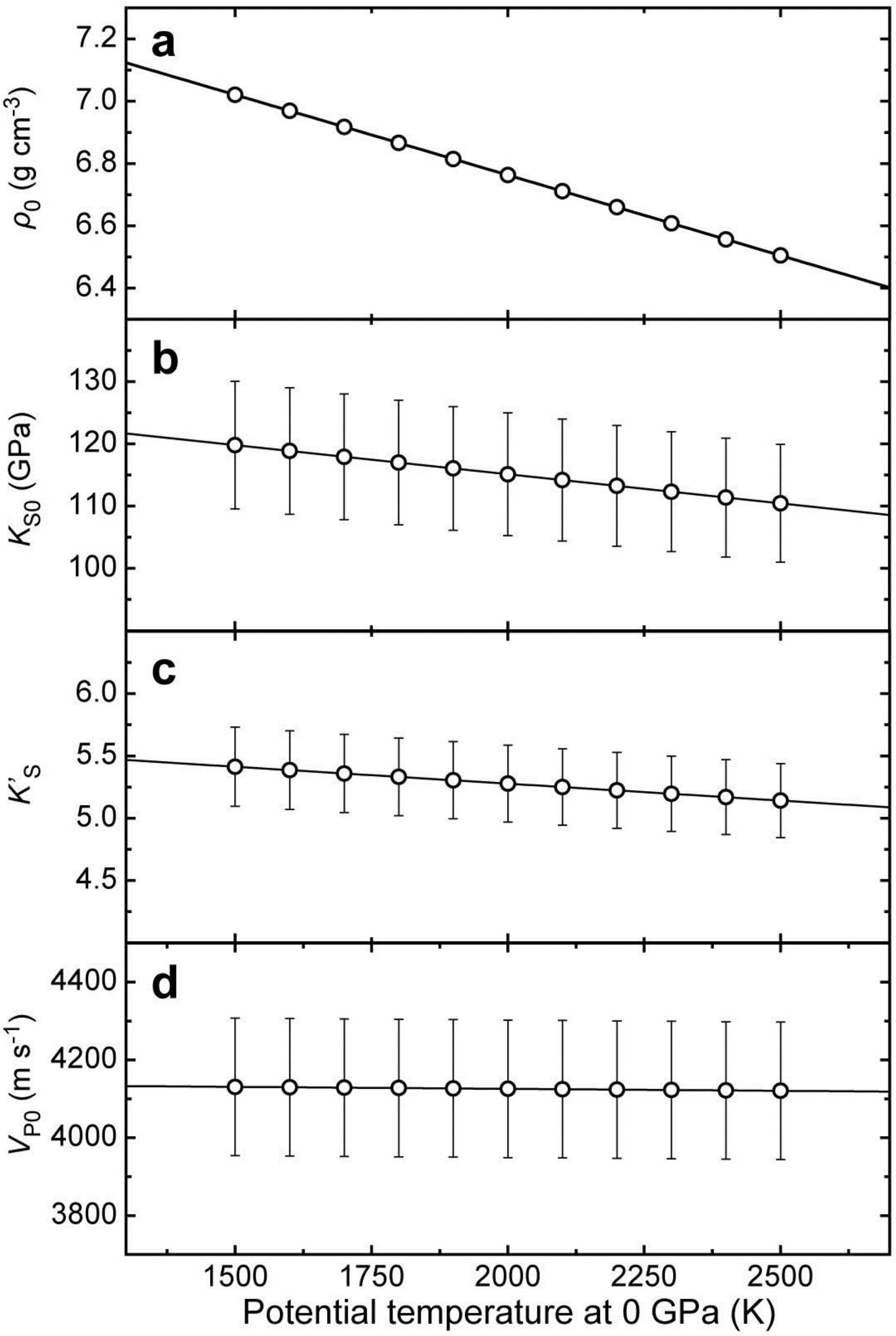

Supplementary Figure 2 | Changes in parameters of the Murnaghan EoS obtained by fitting with a variety of temperature at 1 bar. a, The density at ambient pressure is from ref. 3. The V_P at 1 bar is calculated from K_{S0} and ρ_0.

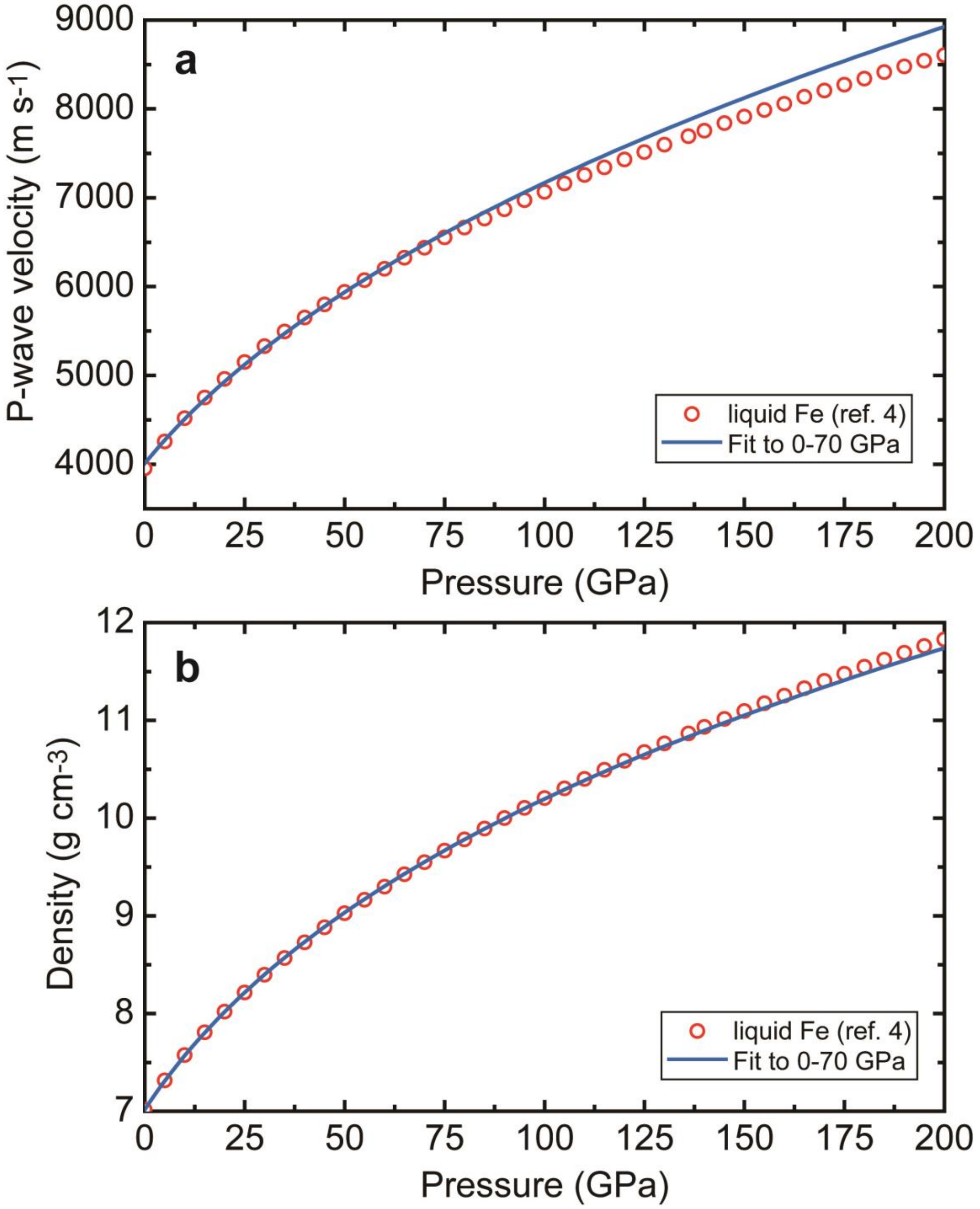

Supplementary Figure 3 | Validity of extrapolation using Murnaghan EoS. The red symbols represent the velocity (**a**) and density (**b**) of liquid Fe along adiabatic compression with T_0 = 1811 K based on the 4th-order Birch-Murnaghan EoS[4]. The extrapolation of the Murnaghan EoS based on fitting to data for 0–70 GPa range (green curve) slightly overestimates the velocity at the core pressure range. See Supplementary Note 1 for details.

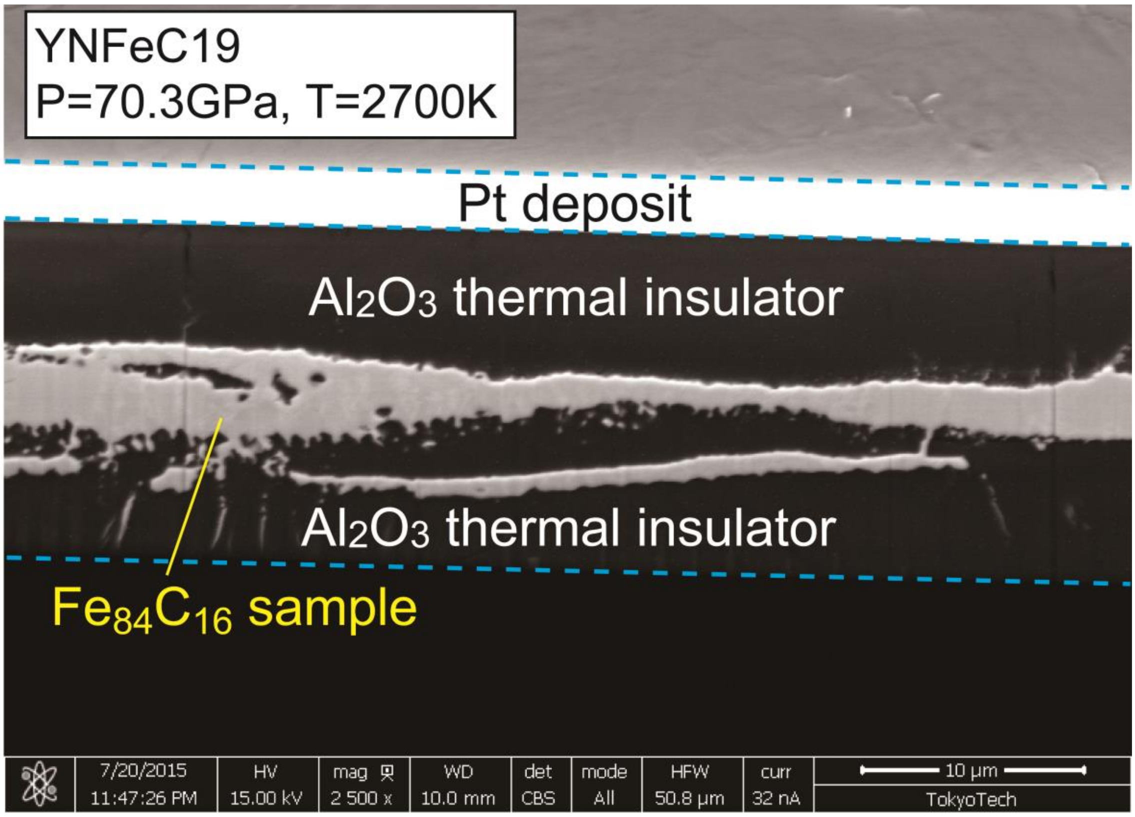

Supplementary Figure 4 | Back-scattered electron image of a sample recovered from 70 GPa and 2700 K. A recovered sample was polished parallel to the compression, laser-heating, and X-ray beam direction using focused ion beam (FIB) instrument. Though a part of liquid sample was penetrated into sapphire thermal insulators, most of sample was kept by sapphire insulators during the heating experiment.

Supplementary Table 1 | Experimental results.

Run no.	*P* (GPa)	*T* (K)	V_P (m s^{-1}) Linear fit*	V_P (m s^{-1}) Sine fit*
FeC08	7.6(1.5)	1480(20)	4592(154)	4766(155)
FeC12	27.3(3.2)	2520(250)	5511(225)	5660(209)
FeC13	26.1(4.0)	2530(250)	5763(263)	5938(155)
FeC14	42.1(4.3)	2850(290)	6485(202)	7169(204)
FeC17	67.8(3.6)	2800(280)	7313(261)	7611(209)
FeC18	30.6(3.1)	2160(220)	6097(294)	6127(204)
FeC19	70.3(2.7)	2700(270)	7283(287)	7771(465)

*The sound velocity of liquid $Fe_{84}C_{16}$ was obtained by linear fit to IXS data. The results of sine fit are for comparison. See Methods for the details.

Supplementary Table 2 | EoS parameters for liquid $Fe_{84}C_{16}$.

T_0 (K)	ρ_0 (g cm^{-3})*	K_{S0} (GPa)	K'_{S0}
2500	6.505	110(9)	5.14(30)
2000	6.763	115(10)	5.28(31)
1500	7.021	120(10)	5.41(32)

*The density for liquid $Fe_{84}C_{16}$ at 1 bar obtained from ref. 3.

Supplementary Note 1: Validity of data extrapolation to core pressures

Our measurements were performed up to 70 GPa and extrapolated to core pressures based on the Murnaghan EoS (see Methods), which considers a simple linear relation between adiabatic bulk modulus and pressure. The validity of such an approximation needs to be verified when we make a large extrapolation[5]. Here we fit the Murnaghan EoS to the limited ranges of $P-V_P$ data (0−70 GPa) for liquid Fe, which are given by the 4th order Birch-Murnaghan EoS along adiabatic temperature profile with T_0 = 1811 K (ref. 4), and then extrapolate to 200 GPa (Supplementary Fig. 3). With fitting parameters same as those obtained for V_P, the density profile is also extrapolated.

We found that the extrapolation of low-pressure (0−70 GPa) data to higher pressure using the Murnaghan EoS overestimates the V_P by 1.5% at 100 GPa and 3.7% at 200 GPa. In contrast, it underestimates the density only by 0.1% at 100 GPa and 0.8% at 200 GPa. It is therefore possible that the present extrapolation of the V_P data of liquid $Fe_{84}C_{16}$ measured up to 70 GPa leads to an overestimation of V_P by 2−4% at the core pressure range (>136 GPa). In addition to the uncertainties of the present EoS parameters (Supplementary Table 2), we incorporated those over- or under-estimations as the uncertainty in the present estimate of carbon abundance in the core. For the static error calculations, a tradeoff between K_{S0} and K'_{S0} was considered.

Supplementary References:

1. Anzellini, S., Dewaele, A., Mezouar, M., Loubeyre, P. & Morard, G. Melting of iron at Earth's inner core boundary based on fast X-ray diffraction. *Science* **340,** 464–466 (2013).

2. Fei, Y. & Brosh, E. Experimental study and thermodynamic calculations of phase relations in the Fe-C system at high pressure. *Earth Planet. Sci. Lett.* **408,** 155–162 (2014).

3. Ogino, K., Nishiwaki, A. & Hosotani, Y. Density of molten Fe-C alloys. *J. Jpn. Inst. Met.* **48,** 1004–1010 (1984).

4. Anderson, W. W. & Ahrens, T. J. An equation of state for liquid iron and implications for the Earth's core. *J. Geophys. Res.* **99,** 4273–4284 (1994).

5. Stacey, F. D. & Davis, P. M. High pressure equations of state with applications to the lower mantle and core. *Phys. Earth Planet. Inter.* **142,** 137–184 (2004).
